# Supplementary material for: Haploinsufficiency of the lysosomal sialidase NEU1 results in a model of pleomorphic rhabdomyosarcoma in mice
Source: Commun Biol. 2022 Sep 20;5:992. doi: 10.1038/s42003-022-03968-8 (PMC9489700; doi:10.1038/s42003-022-03968-8)
Supplement: Supplementary file 2 — Supplementary Information [file 42003_2022_3968_MOESM2_ESM.pdf]

SUPPLEMENTARY INFORMATION

Haploinsufficiency of the lysosomal sialidase *NEU1* results in a model of pleomorphic rhabdomyosarcoma in mice

Eda R. Machado[1‡], Diantha van de Vlekkert[1‡], Heather S. Sheppard[2†], Scott Perry[3†], Susanna M. Downing[4], Jonathan Laxton[3], Richard Ashmun[3], David B. Finkelstein[5], Geoffrey A. Neale[6], Huimin Hu[1], Frank C. Harwood[1], Selene C. Koo[2], Gerard C. Grosveld[1*] & Alessandra d'Azzo[1*].

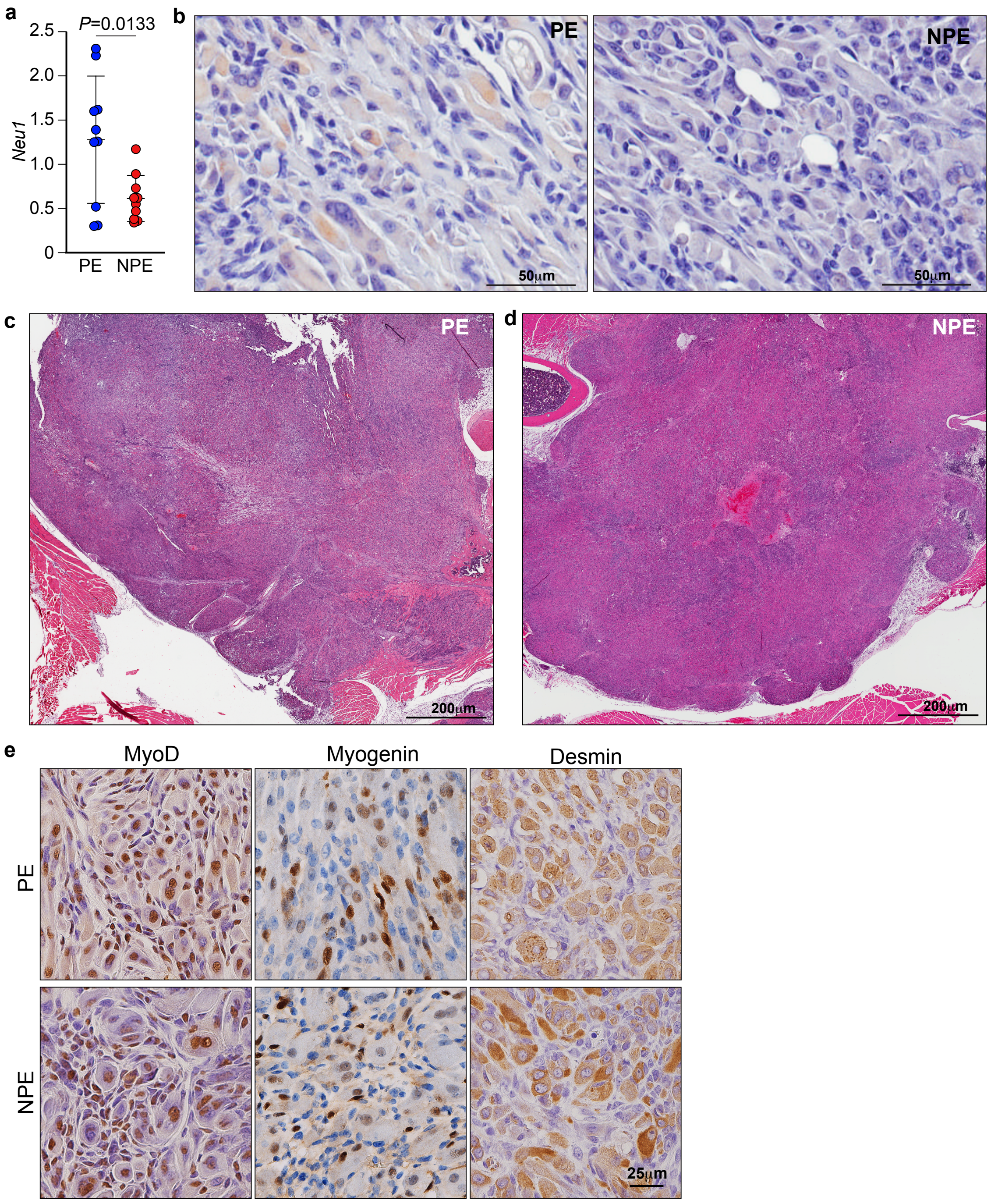

Supplementary Figure 1: Characterization of PE and NPE tumors. a, qRT-PCR analysis of *Neu1* mRNA expression in PE and NPE tumors. Expression is shown as relative to PE. Mean ± s.d.; Welch *t*-test; *n*=10 biologically independent tumors. **b**, Representative micrographs of PE and NPE tumors immunostained for Neu1. Scale bar: 50μm. **c** and **d**, Representative micrographs of H&E-stained overviews of PE (c) and NPE (d) limb tumors. Scale bar: 200μm. **e,** Representative micrographs of PE and NPE tumors immunostained for the RMS markers, MyoD, Myogenin and Desmin. Scale bar: 25μm.

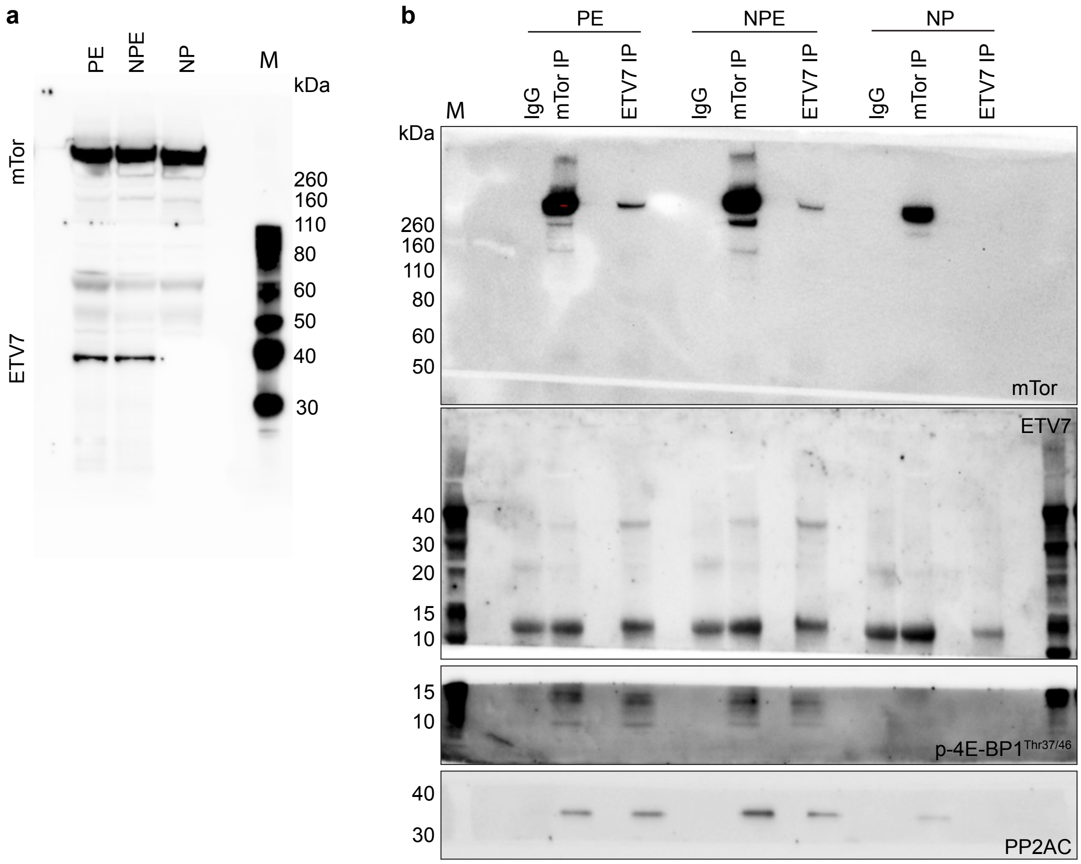

Supplementary Figure 2: mTor and ETV expression in PE and NPE tumors. a,b Uncropped Western blots belonging to Figure 1e and 1f. M: Molecular weight marker. mTor signaling also serves as a loading control, given that the mTor signal does not change.

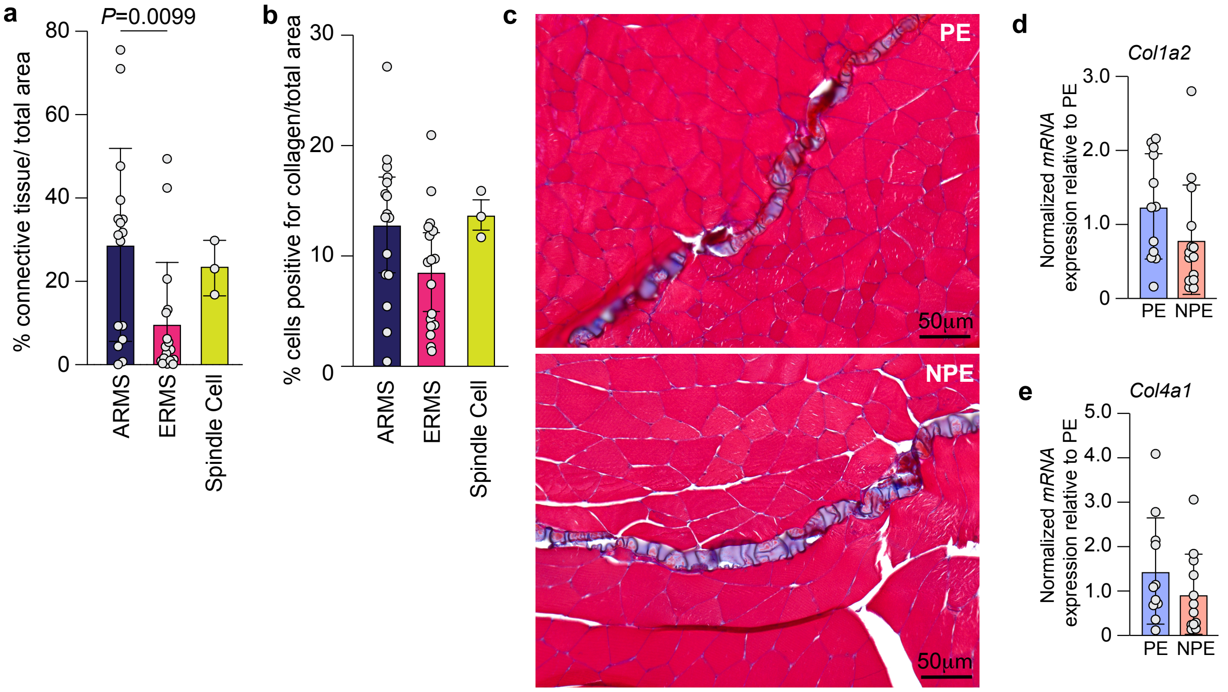

Supplementary Figure 3: Connective tissue expression in normal PE and NPE muscles. a and **b,** Quantification of ECM/collagen deposition (a) and intracytoplasmic (b) in ARMS, ERMS and spindle cell RMS TMA sections. Mean ± s.d.; Welch (unpaired) *t*-test; ARMS: *n*=16; ERMS *n*=17; spindle cell/sclerosing RMS: *n*=3, biologically independent tumors. **c,** Masson's Trichrome staining of normal PE and NPE muscles. Scale bar: 50µm. **d** and **e**, *Col1a2* mRNA expression in normal PE and NPE skeletal muscle. Mean ± s.d.; Welch (unpaired) *t*-test; *n*=12 (PE) and *n*=14 (NPE) biologically independent samples. **g,** *Col4a1* mRNA expression in normal PE and NPE skeletal muscle. Mean ± s.d.; Welch (unpaired) *t*-test; *n*=11 (PE) and *n*=12 (NPE) biologically independent samples.

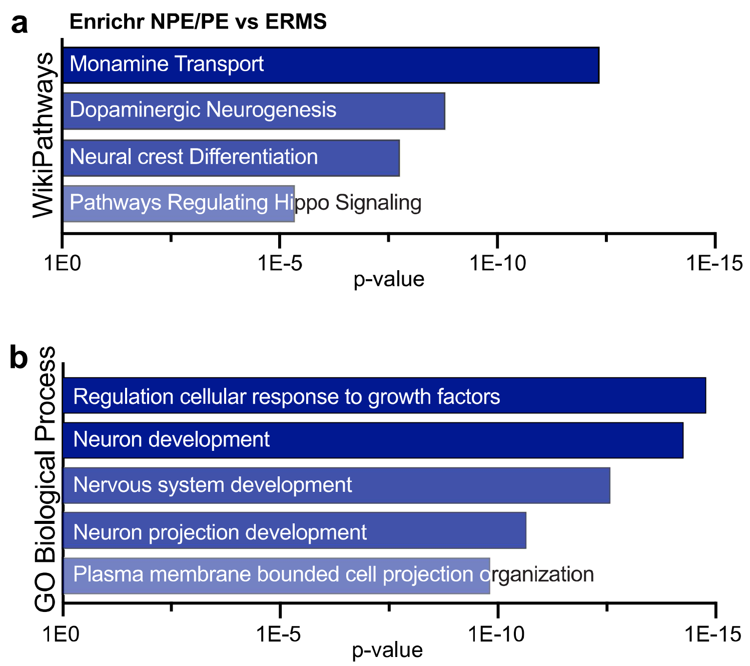

Supplementary Figure 4: Enrichr analysis of PE and NPE gene expression common to human ERMS. a, Murine and human ERMS correlative gene expression enriched in pathways according to the WikiPatways 2021 library; $P \leq 0.05$. **b**, Murine and human ERMS correlative gene expression enriched in pathways defined by the GO Biological Process 2018; $P \leq 0.05$.

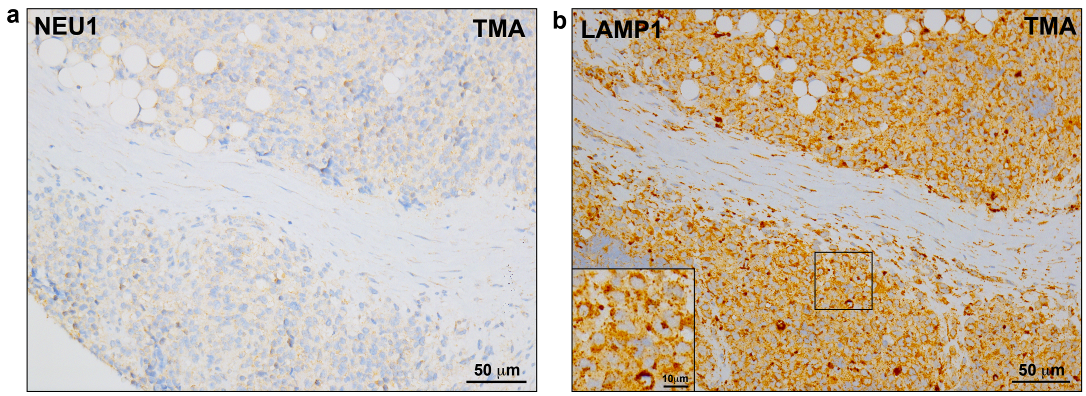

Supplementary Figure 5: Inverse correlation between low NEU1 and high LAMP1 expression in human RMS. a, Low NEU1 and **b,** high LAMP1 immunostaining on a core of RMS TMAs (*n*=41 biologically independent samples). A higher magnification micrograph of the same core shown in Fig. 3. Scale bar: 50µm, inset scale bar: 10µm.

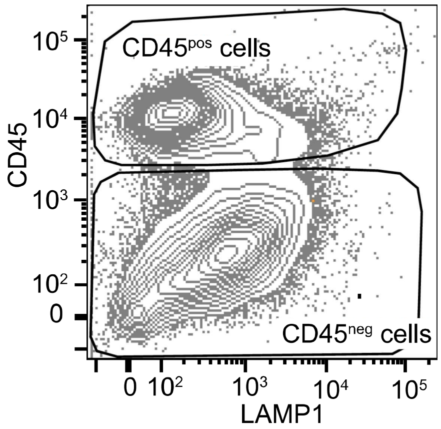

Supplementary Figure 6: Presence of CD45^{pos} and CD45^{neg} cells in murine RMS. Example of gating used to separate CD45^{pos} from CD45^{neg} cells in mouse RMS prior to multiplex flow cytometry analysis.

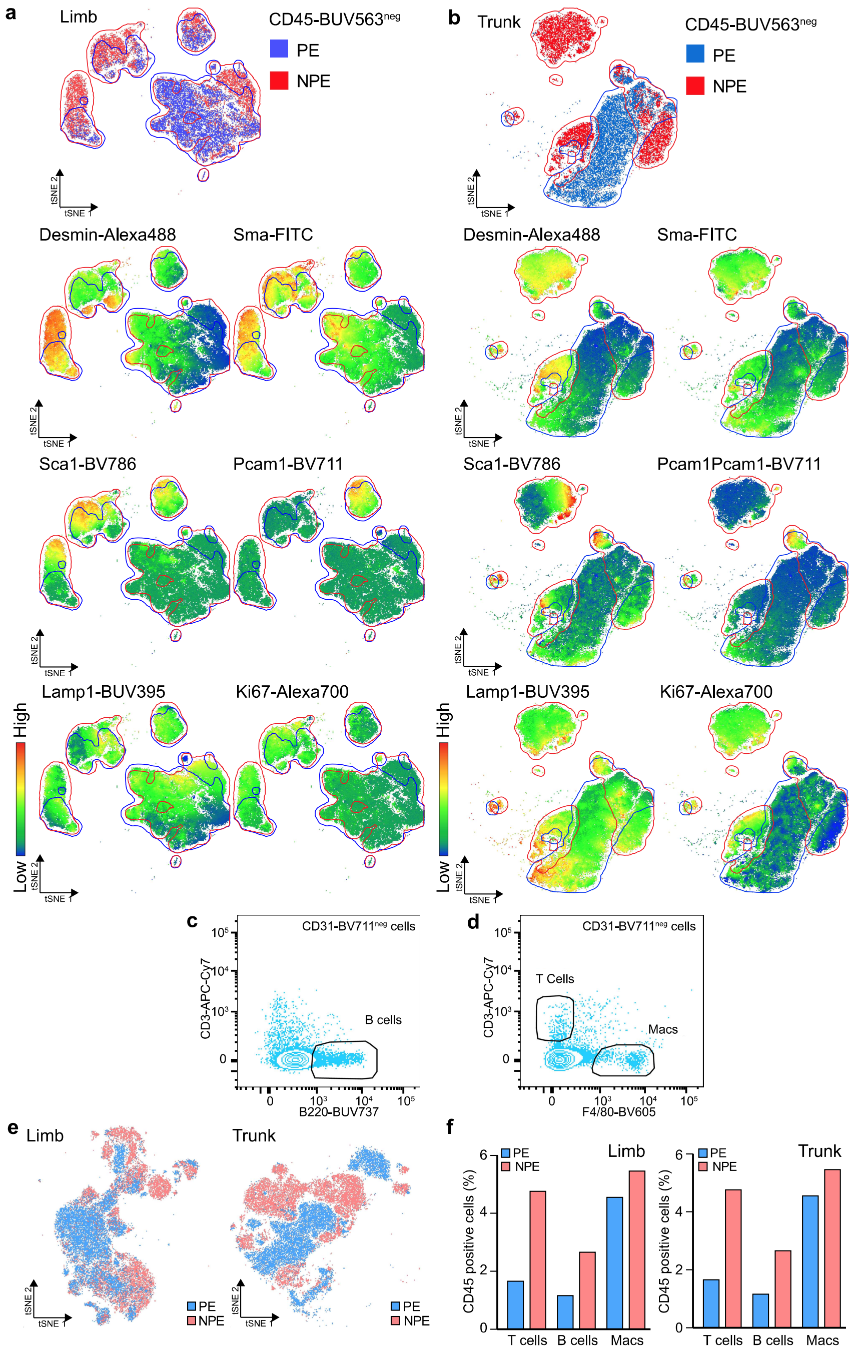

Supplementary Figure 7: Cellular markers that define different populations in NPE and PE RMS. **a** and **b,** tSNE graphs of CD45^{neg} cells from PE and NPE from the limb (a) and trunk (b). Intensity and distribution of each individual marker used are shown throughout the tSNEs. **c,** Example of gating used to separate CD45^{pos} from CD45^{neg} cells in mouse RMS prior to the flow cytometry analysis of CD45^{neg} cells. **d,** Example of gating used to identify T cells and B cells. **e,** tSNE graphs showing the distribution of CD45^{pos} cells from limb and trunk RMS from PE and NPE mice. **f,** Percentage of T cells, B cells and macrophages present in limb and trunk RMS from PE and NPE mice.

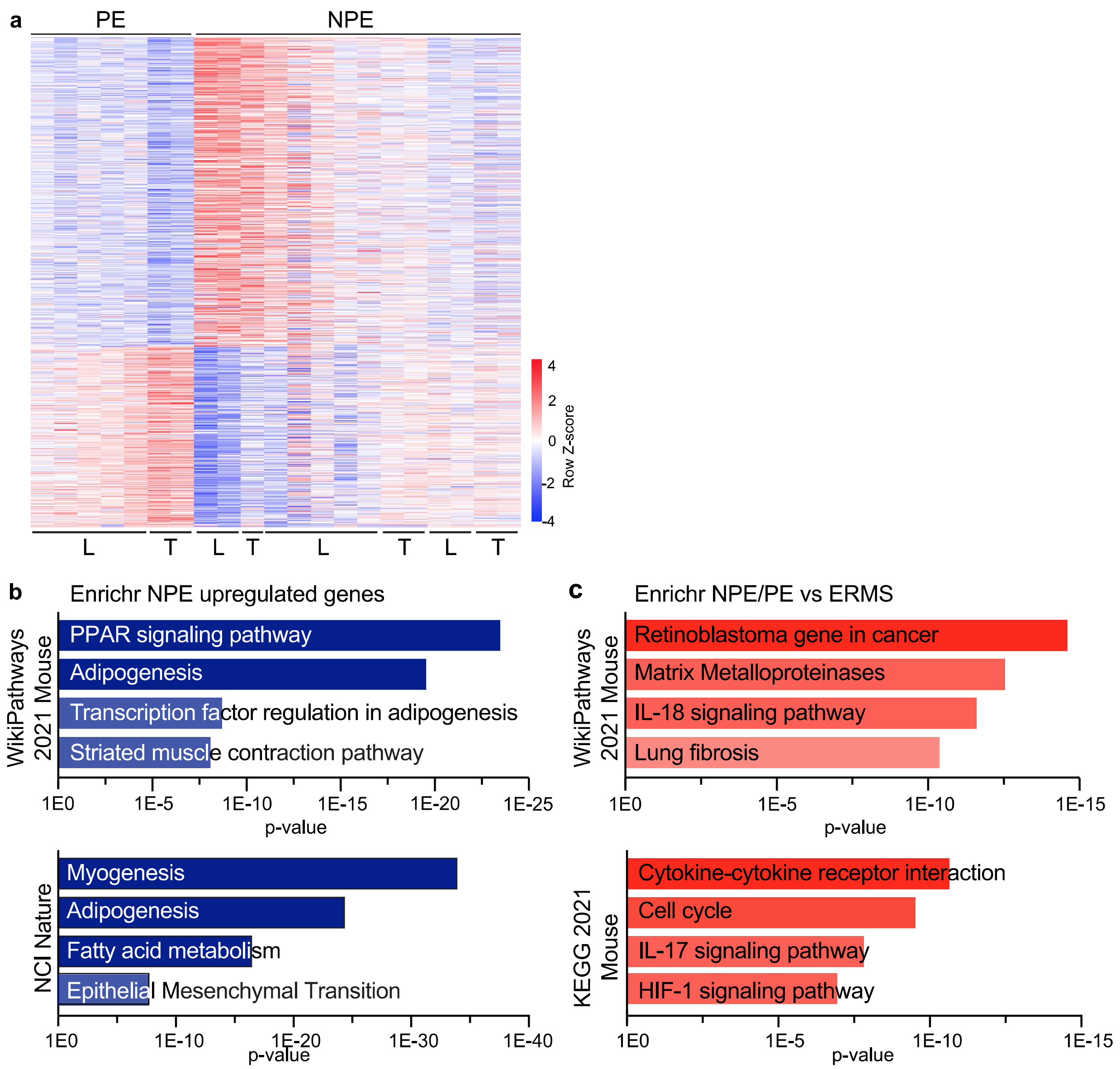

Supplementary Figure 8: Analysis of the genetic landscape of NPE versus PE RMS. a, Heat map of murine microarray data comparing overall mRNA expression between PE (*n*=7 biologically independent tumors) and NPE (*n*=14 biologically independent tumors) RMS samples. A total of 778 genes are shown with $log_2FC \leq -0.5$ and ≥ 0.5; $P \leq 0.5$. L: limb RMS; T: trunk RMS. **b,** Analysis by Encrichr of genes upregulated in NPE compared with PE RMS. Pathways within the Wiki Pathways 2021 and GO Biological Process 2018 libraries are shown in which NPE upregulated genes were enriched; $P < 0.05$. **c,** Analysis by Encrichr of genes downregulated in NPE compared with PE RMS. Pathways within the Wiki Pathways 2021 and GO KEGG 2018 libraries are shown in which NPE downregulated genes were enriched; $P < 0.05$.

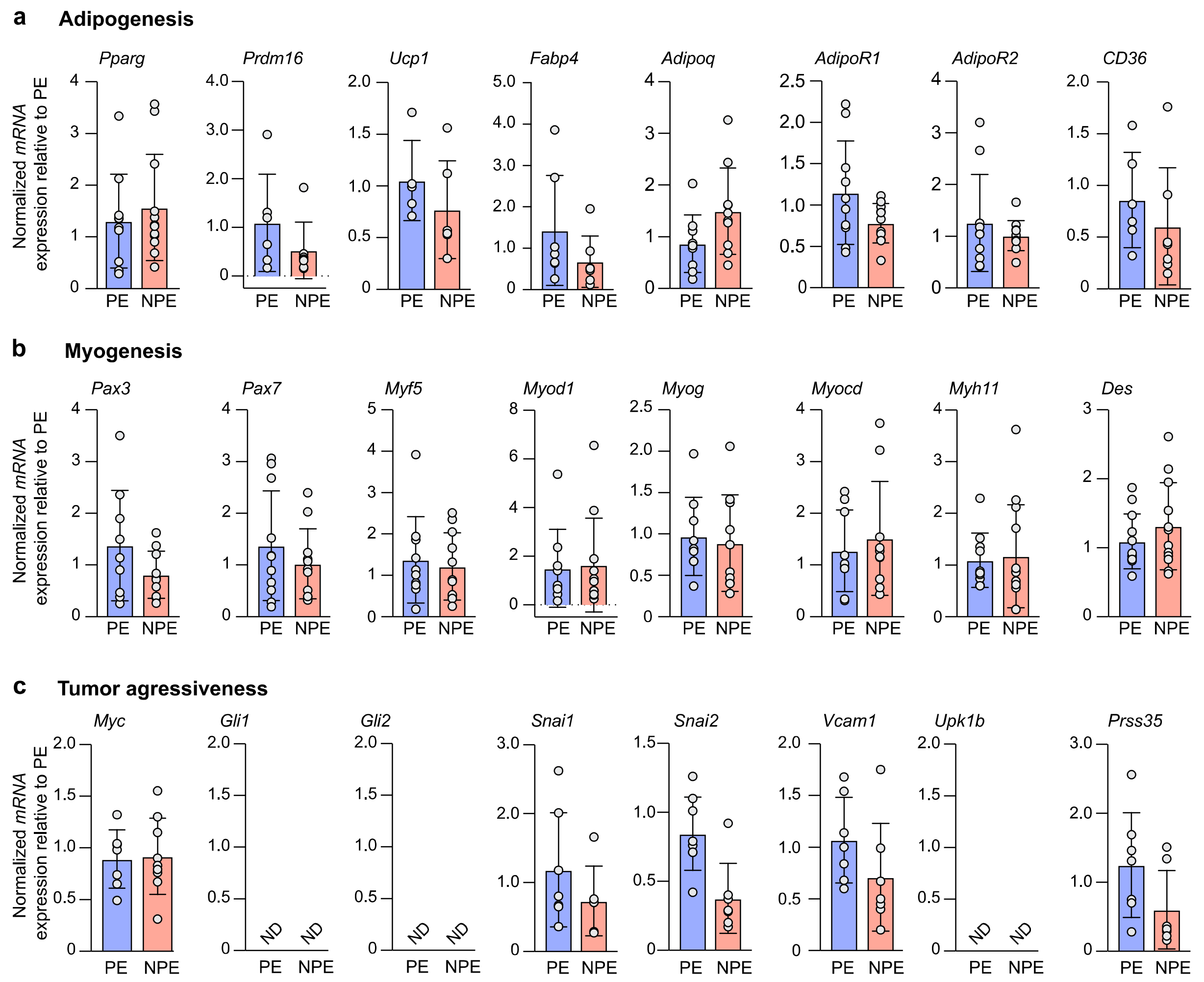

Supplementary Figure 9: Gene expression in normal PE and NPE skeletal muscle. a–c, qRT-PCR analysis of normal PE and NPE skeletal muscle for genes involved in adipogenesis (a); *Pparg* (PE: *n*=10; NPE: *n*=12), *Prdm16* and *CD36* (PE: *n*=6; NPE: *n*=7), *Ucp1* (PE: *n*=5; NPE: *n*=6), *Fabp4* (PE: *n*=7; NPE: *n*=7), *Adipoq* (PE: *n*=9; NPE: *n*=10), *AdipoR1* and *AdipoR2* (PE: *n*=10; NPE: *n*=11), myogenesis (b); *Pax3, Myog* and *Myocd* (PE: *n*=9; NPE: *n*=10), *Pax7* (PE: *n*=11; NPE: *n*=11), *Myf5* (PE: *n*=10; NPE: *n*=11), *Myod1* (PE: *n*=9; NPE: *n*=11), *Myh11* (PE: *n*=10; NPE: *n*=13), *Des* (*n*=12), and tumor aggressiveness (c); *Myc* (PE: *n*=7; NPE: *n*=9), *Snai1* (PE: *n*=7; NPE: *n*=6), *Snai2* (PE: *n*=8; NPE: *n*=7), *Vcam1* and *Prss35* (*n*=7), *Gli1*, *Gli2* and *Upk1b:* ND, not detectable. qRT-PCR results are from normalized *mRNA* expression relative to normal PE muscle. Mean ± s.d.; Welch (unpaired) *t*-test; all biologically independent samples.

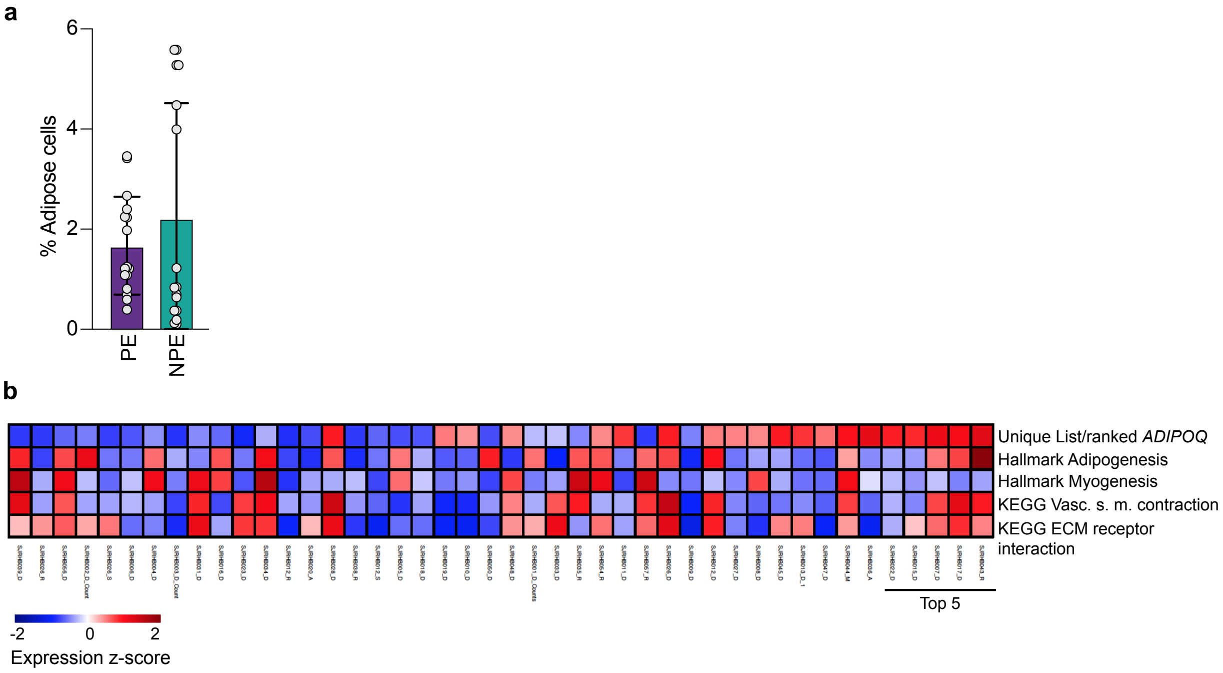

Supplementary Figure 10: GSEA comparison between RMS patients using Hallmark and KEGG pathways. a, Quantification of adipose cell numbers in PE and NPE tumors. Mean ± s.d.; Welch (unpaired) *t*-test. *n*=16 biologically independent samples. **b**, Heat map of *ADIPOQ* expression (unique list) compared with Hallmark Adipogenesis, Hallmark Myogenesis, KEGG Vascular smooth muscle contraction and KEGG ECM receptor interaction. Heat map is ranked by *ADIPOQ* values.

Supplementary Table 1: List of qRT-PCR primers.

Gene	Company	Catalog number	Unique ID	Forward	Reverse
Adipoq	d'Azzo's lab.			GTCAGTGGATCTGACGACACCAA	ATGCCTGCCATCCAACCTG
AdipoR1	d'Azzo's lab.			TCATCTACCTCTCCATCGTCTGTGT	CAAGCCAAGTCCCAGGAACA
AdipoR2	d'Azzo's lab.			TTTGCCACCCCTCAGTATCG	TGACATATTGCAAGGTAGGGATGAT
Des	d'Azzo's lab.			GTGGATGCAGCCACTCTAGC	TTAGCCGCGATGGTCTCATAC
ETV7	d'Azzo's lab.			GTTCTTGTGATTTCCCCAGAGTC	CTGCTGTGGGATTACGTGTATC
Fabp4	d'Azzo's lab.			ATCAGCGTAAATGGGGATTTGG	GTCTGCGGTGATTTCATCGAA
Myc	d'Azzo's lab.			ATGCCCCTCAACGTGAACTTC	GTCGCAGATGAAATAGGGCTG
Myf5	d'Azzo's lab.			GACAGGGCTGTTACATTCAGG	TGAGGGAACAGGTGGAGAAC
Myocd	d'Azzo's lab.			ACACTCCTGGGGTCTGAACA	GCGGTATTAAGCCTTGGTTAGC
Myod1	d'Azzo's lab.			CGGGACATAGACTTGACAGGC	TCGAAACACGGGTCATCATAGA
Myog	d'Azzo's lab.			CGATCTCCGCTACAGAGGC	GTTGGGACCGAACTCCAGT
Pax3	d'Azzo's lab.			CATCCGACCTGGTGCCATC	ATTTCCCAGCATAACATGCCC
Pax7	d'Azzo's lab.			GTCGGGTTCTGATTCCACAT	GCGAGAAGAAAGCCAAACAC
Ppary	d'Azzo's lab.			CTCCAAGAATACCAAAGTGCGA	GCCTGATGCTTTATCCCCACA
Prss35	d'Azzo's lab.			CCCTCTCTGATGGGTCGGAAA	TTCCTGACATTCGATGCCACA
Snai1	d'Azzo's lab.			CACACGCTGCCTTGTGTCT	GGTCAGCAAAAGCACGGTT
Snai2	d'Azzo's lab.			TGGTCAAGAAACATTTCAACGCC	GGTGAGGATCTCTGGTTTTGGTA
Upk1b	d'Azzo's lab.			CACTGTTCGTTGCTTCCAGG	GCTTCGAGAAGTGGGTAAAGACT
Vcam1	d'Azzo's lab.			AGTTGGGGATTCGGTTGTTC	CATTCCTTACCACCCCATTG
Col1a2	QIAGEN	PPM04448F			
Col4a1	QIAGEN	PPM05145A			
Gli1	Biorad	10025636	qMmuCID0026119		
Gli2	Biorad	10041595	qMmuCID0005725		
Myh11	QIAGEN	PPM04496A			
Neu1	BioRad	10025636	qMmuCID0020645		
Prdm16	Biorad	1004195	qMmuCID0010482		
Ucp1	Biorad	10041595	qMmuCID0005832		

Supplementary Table 2: List of antibodies.

Antibody	Vendor	Cat#	Usage	Dilution
mTOR (L27D4)	Cell Signaling Technologies	4517S	WB	1:2000
mTOR (N-19)	Santa Cruz	sc1549	IP	1:2000
(p-)4EBP1Thr37/46 (236B4)	Cell Signaling Technologies	2855	WB	1:2000
ETV7 (7E4)	Dr. Grosveld's lab.		IP	1:2000
ETV7	Sigma	HPA 029033	WB	1:2000
PP2Ac (1D6)	Upstate biotechnology	05-421	WB	1:2000
MyoD (G-1)	Santa Cruz Biotechnology	sc-377460	IHC	1:300
Myogenin (F5D)	Cell Marque	296M-14	IHC	1:100
Desmin (Y66)	Abcam	ab32362	IHC	1:1000
Adiponectin (EPR17019)	Abcam	ab181281	IHC	1:4000
Adiponectin	Abcam	ab216502	IHC	1:150
NEU1	Dr. d'Azzo's lab.		IHC	1:50
LAMP1 (C54H11)	Cell Signaling Technologies	3243	IHC	1:1000
CD107a (LAMP1)-BUV395 (1D4B)	BD Bioscience	565533	Fcyt	1:20
CD45-BUV563 (30-F11)	BD Bioscience	565710	Fcyt	1:100
B220-BUV737 (RA3-6B2)	BD Bioscience	564449	Fcyt	1:50
CD44-BV421 (IM7)	BD Bioscience	563970	Fcyt	1:50
F4/80-BV605 (T45-2342)	BD Bioscience	743281	Fcyt	1:20
CD31 (PCAM1)-BV711 (MEC13.3)	BD Bioscience	740680	Fcyt	1:50
Sca1-BV786 (D7)	BD Bioscience	563991	Fcyt	1:50
CD326(EpCAM)-PE-Cy7 (G8.8)	eBioscience	25-5791-80	Fcyt	1:50
Lyve1-e660 (ALY7)	eBioscience	50-0443-80	Fcyt	1:50
CD3-APC-Cy7 (145-2C11)	BD Bioscience	557596	Fcyt	1:40
Desmin-Alexa488 (Y66)	Abcam	ab185033	Fcyt	1:40
SMA-FITC	Sigma-Aldrich	F3777	Fcyt	1:70
Ki67-Alexa700	Biolegend	652420	Fcyt	1:50
FSP1-S100A4-PerCP-Cy5.5	Biolegend	370009	Fcyt	1:20

WB: Western Blot; IP: immunoprecipitation; IHC: immunohostochemistry; Fcyt: flow cytometry
