# Supplementary material for: Haploinsufficiency of the lysosomal sialidase NEU1 results in a model of pleomorphic rhabdomyosarcoma in mice
Source: Commun Biol. 2022 Sep 20;5:992. doi: 10.1038/s42003-022-03968-8 (PMC9489700; doi:10.1038/s42003-022-03968-8)
Supplement: Supplementary file 3 — Description of Additional Supplementary Files [file 42003_2022_3968_MOESM3_ESM.pdf]

Description of Additional Supplementary Files

File name: Supplementary Data 1
Description: The source data behind the graphs in the paper.

File name: Supplementary Data 2
Description: Enrichr analysis of genes expressed in NPE and PE tumors that correlate with those expressed in ERMS ($P \leq 0.05$).

File name: Supplementary Data 3
Description: Up- and downregulated genes in NPE vs PE RMS (log2FC ≤ -0.5 and ≥ 0.5) $P \leq 0.5$.

File name: Supplementary Data 4
Description: Enrichr analysis of genes upregulated in NPE RMS versus those in PE RMS ($P < 0.05$).

File name: Supplementary Data 5
Description: Enrichr analysis of genes downregulated in NPE RMS versus those in PE RMS ($P < 0.05$).

File name: Supplementary Data 6
Description: GSEA of top ranked differentially expressed genes in NPE versus PE RMS.

File name: Supplementary Data 7
Description: Comparison of gene expression profiles in RMS patients with gene expression of MSigDB pathways. Results are ranked by ADIPOQ expression.
