# Supplementary material for: Haploinsufficiency of the lysosomal sialidase NEU1 results in a model of pleomorphic rhabdomyosarcoma in mice
Source: Commun Biol. 2022 Sep 20;5:992. doi: 10.1038/s42003-022-03968-8 (PMC9489700; doi:10.1038/s42003-022-03968-8)
Supplement: Supplementary file 11 — Reporting Summary [file 42003_2022_3968_MOESM11_ESM.pdf]

Corresponding author(s): Dr. Gerard C. Grosveld and Dr. Alessandra d'Azzo

Last updated by author(s): Aug 31, 2022

Reporting Summary

Nature Portfolio wishes to improve the reproducibility of the work that we publish. This form provides structure for consistency and transparency in reporting. For further information on Nature Portfolio policies, see our Editorial Policies and the Editorial Policy Checklist.

Statistics

For all statistical analyses, confirm that the following items are present in the figure legend, table legend, main text, or Methods section.

n/a	Confirmed	
☐	☒	The exact sample size (n) for each experimental group/condition, given as a discrete number and unit of measurement
☐	☒	A statement on whether measurements were taken from distinct samples or whether the same sample was measured repeatedly
☐	☒	The statistical test(s) used AND whether they are one- or two-sided<br>*Only common tests should be described solely by name; describe more complex techniques in the Methods section.*
☒	☐	A description of all covariates tested
☒	☐	A description of any assumptions or corrections, such as tests of normality and adjustment for multiple comparisons
☐	☒	A full description of the statistical parameters including central tendency (e.g. means) or other basic estimates (e.g. regression coefficient) AND variation (e.g. standard deviation) or associated estimates of uncertainty (e.g. confidence intervals)
☒	☐	For null hypothesis testing, the test statistic (e.g. F, t, r) with confidence intervals, effect sizes, degrees of freedom and P value noted<br>*Give P values as exact values whenever suitable.*
☒	☐	For Bayesian analysis, information on the choice of priors and Markov chain Monte Carlo settings
☒	☐	For hierarchical and complex designs, identification of the appropriate level for tests and full reporting of outcomes
☐	☒	Estimates of effect sizes (e.g. Cohen's d, Pearson's r), indicating how they were calculated

Our web collection on statistics for biologists contains articles on many of the points above.

Software and code

Policy information about availability of computer code

Data collection

For this study no custom code was used to collect data.
Trichrome Masson's and H&E stained slides were scanned with Aperio Scanscope XT (Leica Biosystems, Inc)
IHC stained slides from RMS TMAs were scanned with the Panoramic 250 Flash III (3DHistech, Inc.).
Microarray cRNA was hybridized to Clariom S Mouse GeneChip (Affymetrix Inc) and signals summarized by RMA (Affymetrix Expression Console v1.1).
Patient's RMS gene expression data was obtained from the Pediatric Cancer Genome Project (PCGP) RNAseq and from public RMS data (PeCan; https://pecan.stjude.cloud/home). Additional gene sets for transcriptional analysis were identified using Enrichr.

Data analysis

For this study no custom code was used to analyze the data.
Aperio color deconvolution and colocalization algorithms and ImageScope software v12.4.3 (Leica Biosystems, Inc.) were used in Trichrome Massons's stained slides.
Nuclear morphometry was analyzed on H&E slides using the ImageScope software v12.4.3 (Leica Biosystems Inc). The Genie tissue classifier software was trained to identify and quantify tumor nuclei.
IHC slides were analyzed with HALO v3.2.1851.354 software (Indica Labs) and Membrane v1.7 algorithm and spacial Analysis Module HALO 3.2 was also used.
Microarray probe signals were transformed into log2 with the Robust Multiarray Average algorithm (Partek Genomics Suite 6.6).
DAVID bioinformatics databases (http://david.abcc.ncifcrf.gov/) was used for functional enrichment analysis of gene lists.
Voom-lima in R (version 4.2) was used to analyze adipogenic gene expression and microarray.
Enrichr (https://maayanlab.cloud/Enrichr/) was used to analyze genetic pathways.
Self-organizing maps and tSNEs for flow cytometry data were analyzed with FLOWSOM. FlowJo and FACS Diva software suites were also used.

Correlations were calculated with Pearson's correlation of deciles of the mean log2 FPKM (for RNA seq) and RMA values for the microarray's expression by class. GSEA (version 3.0) (https://www.gsea-msigdb.org/gsea/index.jsp) was performed using the curated pathways from MSigDB.
Differentially expressed transcripts were identified by ANOVA and the FDR was estimated.
RT-qPCR data was normalized to 18S ribosomal RNA and plotted in Prism 9.
All detailed software and usage is described in Material and Methods.

For manuscripts utilizing custom algorithms or software that are central to the research but not yet described in published literature, software must be made available to editors and reviewers. We strongly encourage code deposition in a community repository (e.g. GitHub). See the Nature Portfolio guidelines for submitting code & software for further information.

Data

Policy information about availability of data

All manuscripts must include a data availability statement. This statement should provide the following information, where applicable:

- Accession codes, unique identifiers, or web links for publicly available datasets
- A description of any restrictions on data availability
- For clinical datasets or third party data, please ensure that the statement adheres to our policy

DATA AVAILABILITY
The data that support the findings of this study is available from the corresponding authors on reasonable request. Uncropped and unedited blot images are provided in Supplementary Figure 2. Microarray data in this publication are deposited in NCBI's functional genomic data repository Gene Expression Omnibus (GEO) and are assecible through GEO series accession number GSE212378. Previously published datasets used in this study were deposited in the European Bioinformatics Institue (EMBL-EBI) and assecible through accession number EGAS00001000256. Source data underlying Figs. 1b, d, I, j, 2d-g, 3d, g, h, 6c-e, 7c-l and Supplementary Fig. 1a, 3a, b, d, e, 9a-c, 10a are provided within this paper (Supplementary Data 1). Patient's RMS gene expression data can be accessed through StJude open resource page (PeCan https://pecan.stjude.cloud/home).

Human research participants

Policy information about studies involving human research participants and Sex and Gender in Research.

Reporting on sex and gender	NA
Population characteristics	NA
Recruitment	NA
Ethics oversight	NA

Note that full information on the approval of the study protocol must also be provided in the manuscript.

Field-specific reporting

Please select the one below that is the best fit for your research. If you are not sure, read the appropriate sections before making your selection.

☒ Life sciences ☐ Behavioural & social sciences ☐ Ecological, evolutionary & environmental sciences

For a reference copy of the document with all sections, see nature.com/documents/nr-reporting-summary-flat.pdf

Life sciences study design

All studies must disclose on these points even when the disclosure is negative.

Sample size	Samples sizes were chosen based on availability of material, taking into consideration n>6 for the best statistical power.
Data exclusions	PCA and quality control metrics removed outliers from both PCGP RNAseq and murine microarray data.
Replication	We confirm that all attempts for replication were successful, when available. Four or more independent analyses were used when possible and indicated in the figure legend, unless unique tumor samples were used that could not be replicated. Two way-co IP with ETV7 in tumors samples was done twice due to limited sample amount.
Randomization	Randomization is not applicable to this study as this is to compare different genetic background mice.
Blinding	Pathologist was blinded to the data when analyzing histological slides.

Reporting for specific materials, systems and methods

We require information from authors about some types of materials, experimental systems and methods used in many studies. Here, indicate whether each material, system or method listed is relevant to your study. If you are not sure if a list item applies to your research, read the appropriate section before selecting a response.

Materials & experimental systems

n/a	Involved in the study
☐	☒ Antibodies
☒	☐ Eukaryotic cell lines
☒	☐ Palaeontology and archaeology
☐	☒ Animals and other organisms
☒	☐ Clinical data
☒	☐ Dual use research of concern

Methods

n/a	Involved in the study
☒	☐ ChIP-seq
☐	☒ Flow cytometry
☒	☐ MRI-based neuroimaging

Antibodies

Antibodies used

mTOR (L27D4), Cell Signaling Technologies, 4517S, WB
mTOR (N-19), Santa Cruz, sc1549, IP
(p-) 4EBP1Thr37/46 (236B4), Cell Signaling Technologies, 2855, WB
ETV7 (7E4), Dr. Grosveld's lab., IP
ETV7, Sigma, HPA 029033, WB
PP2Ac (1D6), Upstate biotechnology, 05-421, WB
MyoD (G-1), Santa Cruz Biotechnology, sc-377460, IHC
Myogenin (F5D), Cell Marque, 296M-14, IHC
Desmin (Y66), Abcam, ab32362, IHC
Adiponectin (EPR17019), Abcam, ab181281, IHC
Adiponectin, Abcam, ab216502, IHC
NEU1, Dr. d'Azzo's lab., IHC
LAMP1 (C54H11), Cell Signaling, 3243, IHC
CD107a (LAMP1)-BUV395 (1D4B), BD Bioscience, 565533, Flow cytometry
CD45-BUV563 (30-F11), BD Bioscience, 565710, Flow cytometry
B220-BUV737 (RA3-6B2), BD Bioscience, 564449, Flow cytometry
CD44-BV421 (IM7), BD Bioscience, 563970, Flow cytometry
F4/80-BV605 (T45-2342), BD Bioscience, 743281, Flow cytometry
CD31 (PCAM1)-BV711 (MEC13.3), BD Bioscience, 740680, Flow cytometry
Sca1-BV786 (D7), BD Bioscience, 563991, Flow cytometry
CD326(EpCAM)-PE-Cy7 (G8.8), eBioscience, 25-5791-80, Flow cytometry
Lyve1-e660 (ALY7), eBioscience, 50-0443-80, Flow cytometry
CD3-APC-Cy7 (145-2C11), BD Bioscience, 557596, Flow cytometry
Desmin-Alexa488 (Y66), Abcam, ab185033, Flow cytometry
SMA-FITC, Sigma-Aldrich, F3777, Flow cytometry
Ki67-Alexa700, Biolegend, 652420, Flow cytometry
FSP1-S100A4-PerCP-Cy5.5, Biolegend, 370009, Flow cytometry

Validation

mTOR (L27D4) https://www.cellsignal.com/products/primary-antibodies/mtor-l27d4-mouse-mab/4517?site-search-type=Products&N=4294956287&Ntt=4517s&fromPage=plp&_requestid=575956
mTOR (N-19) https://www.scbt.com/p/mtor-antibody-n-19?requestFrom=search
(p)4EBP1Thr37/47 (236B4) https://www.cellsignal.com/products/primary-antibodies/phospho-4e-bp1-thr37-46-236b4-rabbit-mab/2855
ETV7 (7E4) in house antibody validated in previous studies (Harwood et al., 2018 Science Advances)
ETV7 https://www.sigmaaldrich.com/US/en/product/sigma/hpa029033
PP2Ac (1D6) https://www.sigmaaldrich.com/US/en/product/mm/05421
MyoD (G1) https://www.scbt.com/p/myod-antibody-g-1?requestFrom=search
Myogenin (F5D) https://www.cellmarque.com/antibodies/CM/119/Myogenin_F5D
Desmin (Y66) https://www.abcam.com/desmin-antibody-y66-cytoskeleton-marker-ab32362.html
Adiponectun (EPR17019) https://www.abcam.com/adiponectin-antibody-epr17019-ab181281.html
Adiponectin https://www.abcam.com/adiponectin-antibody-ab216502.html
NEU1 in house antibody validated in previous studies (Mosca et al., 2020 J. Clin. Med.)
Lamp1 (C54H11) https://www.cellsignal.com/products/primary-antibodies/lamp1-c54h11-rabbit-mab/3243
CD107a (LAMP1)-BUV395 (1D4B) https://www.bdbiosciences.com/en-us/products/reagents/flow-cytometry-reagents/research-reagents/single-color-antibodies-ruo/buv395-rat-anti-mouse-cd107a.565533
CD45-BUV563 (30-F11) https://www.bdbiosciences.com/en-us/products/reagents/flow-cytometry-reagents/research-reagents/single-color-antibodies-ruo/buv563-rat-anti-mouse-cd45.612924
B220-BUV737 (RA3-6B2) https://www.bdbiosciences.com/en-us/products/reagents/flow-cytometry-reagents/research-reagents/single-color-antibodies-ruo/buv737-rat-anti-mouse-cd45r-b220.612838
CD44-BV421 (IM7) https://www.bdbiosciences.com/en-us/products/reagents/flow-cytometry-reagents/research-reagents/single-color-antibodies-ruo/bv421-rat-anti-mouse-cd44.563970

F4/80-BV605 (T45-2342) https://www.bdbiosciences.com/en-us/products/reagents/flow-cytometry-reagents/research-reagents/single-color-antibodies-ruo/bv605-rat-anti-mouse-f4-80.743281
CD31 (PCAM1)-BV711 (MEC13.3) https://www.bdbiosciences.com/en-us/products/reagents/flow-cytometry-reagents/research-reagents/single-color-antibodies-ruo/bv711-rat-anti-mouse-cd31.740680
Sca1-BV786 (D7) https://www.bdbiosciences.com/en-us/products/reagents/flow-cytometry-reagents/research-reagents/single-color-antibodies-ruo/bv786-rat-anti-mouse-ly-6a-e.563991
CD326(EpCAM)-PE-Cy7 (G8.8) https://www.thermofisher.com/antibody/product/CD326-EpCAM-Antibody-clone-G8-8-Monoclonal/25-5791-80
Lyve1-e660 (ALY7) https://www.thermofisher.com/antibody/product/LYVE1-Antibody-clone-ALY7-Monoclonal/50-0443-80
CD3-APC-Cy7 (145-2C11) https://www.bdbiosciences.com/en-us/products/reagents/flow-cytometry-reagents/research-reagents/single-color-antibodies-ruo/apc-cy-7-hamster-anti-mouse-cd3e.557596
Desmin-Alexa488 (Y66) https://www.abcam.com/alexa-fluor-488-desmin-antibody-y66-cytoskeleton-marker-ab185033.html
SMA-FITC https://www.sigmaaldrich.com/US/en/product/sigma/f3777 (also tested by StJude Flow Cytometry Core)
Ki67-Alexa700 https://www.biolegend.com/en-us/products/alexa-fluor-700-anti-mouse-ki-67-antibody-10366
FSP1-S100A4-PerCP-Cy5.5 https://www.biolegend.com/en-us/products/percp-cyanine5-5-anti-human-s100a4-antibody-13120

Animals and other research organisms

Policy information about studies involving animals; ARRIVE guidelines recommended for reporting animal research, and Sex and Gender in Research

Laboratory animals	Neu1+/– FVB/NJ69 mice were crossed with Ptch1+/–/ETV7TG/+/– (129sv/C57BL/6) mice. The mice used in this study were obtained over a timespan of 2 years.
Wild animals	This study did not involve wild animals.
Reporting on sex	Sex-based analysis was not considered, all animals that developed tumors were analyzed independently of gender. Genetic microarray analysis of genetic landscape based on sex did not find any statistical difference between male and female mice.
Field-collected samples	This study did not involve samples collected from the field.
Ethics oversight	All procedures were performed following NIH guidelines and animal protocols approved by the St Jude Children's Research Hospital Institutional Animal Care and Use Committee (IACUC). The Institutional Review board (IRB) approved the use of all human tumor samples/slides.

Note that full information on the approval of the study protocol must also be provided in the manuscript.

Flow Cytometry

Plots

Confirm that:

☒ The axis labels state the marker and fluorochrome used (e.g. CD4-FITC).

☒ The axis scales are clearly visible. Include numbers along axes only for bottom left plot of group (a 'group' is an analysis of identical markers).

☒ All plots are contour plots with outliers or pseudocolor plots.

☒ A numerical value for number of cells or percentage (with statistics) is provided.

Methodology

Sample preparation	NPE and PE tumors were dissociated using the Mouse Dissociation kit and Gentle MACS Octo Dissociator, following the manufacturer's instructions (MACS, Miltenyi Biotec). Large aggregates persisting in the cell preparation were then removed by filtering them through 70 µm cell strainers. The resulting single-cell suspensions were stained with fluorochrome-conjugated monoclonal antibodies before analyses.
Instrument	BD FACSymphony A9 analyzer (BD, San Jose) equipped with 355nm, 400nm, 440nm, 488nm, 561nm, and 640nm lasers for excitation and an array of 30 detectors with the appropriate light filters for resolving the specified fluorochromes.
Software	Self-organizing maps for visualizing and interpreting cytometry data, FlowSOM, was used on 60 000 cells from limb and trunk NPE and PE RMS to generate density, heat-maps and tSNE graphs. Additional two-dimensional analyses were conducted using both FlowJo and FACS Diva software suites.
Cell population abundance	Cellular clusters were identified and annotated based on the differential expression of markers.
Gating strategy	Cells from dissociated PE and NPE tumors.<br>Non-hematopoietic cells:<br>1. SSC and FSC<br>2. CD45-BUV563neg and CD3-APC-Cy7neg

3. CD107-BUV396pos
3.1 Epithelial cells: CD326-PE-Cy7pos and CD31-BV711neg
3.2 Endothelial cells: CD326-PE-Cy7neg and CD31-BV711pos
3.3 Lymphatics: Lyve1-e660pos and CD31-BV711neg
3.4 Blood vessels: Lyve1-e660neg and CD31-BV711pos
3.5 Stem cells: Sca1-BV786pos and Ki67-Alexa700neg
3.6 Stem cells (proliferation): Sca1-BV786pos and Ki67-Alexa700pos
3.7 Proliferating: Sca1-BV786neg and Ki67-Alexa700pos

Hematopoietic cells:
1. SSC and FSC
2. CD45-BUV563pos and CD3-APC-Cy7pos
3. CD107-BUV396pos
3.1 B-cells: B220-BUV737pos and F4/80-BV605neg
3.2 T-cells: CD3-APC-Cy7pos and F4/80-BV605neg
3.3 Macs: CD3-APC-Cy7neg and F4/80-BV605pos

Tumor/stromal cells:
1. SSC and FSC
2. CD45-BUV563pos
2.1 CD31-BV711pos and CD107-BUV396pos
2.2 Sca1-BV786pos and CD107-BUV396pos
2.3 SMA-Alexa488pos and CD107-BUV396pos
2.4 FSP1-PerCP-Cy5.5pos and CD107-BUV396pos
2.5 Desmin-PEpos and CD107-BUV396pos
2.6 CD326-PE-Cy7pos and CD107-BUV396pos
2.7 Lyve1-e660pos and CD107-BUV396pos
2.8 Ki67-Alexa700pos and CD107-BUV396pos

☒ Tick this box to confirm that a figure exemplifying the gating strategy is provided in the Supplementary Information.
